# Synchronous and Time-Dependent Expression of Cyclins, Cyclin-Dependant Kinases, and Apoptotic Genes in the Rumen Epithelia of Butyrate-Infused Goats

**DOI:** 10.3389/fphys.2018.00496

**Published:** 2018-05-23

**Authors:** Jamila Soomro, Zhongyan Lu, Hongbing Gui, Bei Zhang, Zanming Shen

**Affiliations:** ^1^Key Laboratory of Animal Physiology and Biochemistry, College of Veterinary Medicine, Nanjing Agricultural University, Nanjing, China; ^2^Department of Veterinary Physiology and Biochemistry, Faculty of Animal Husbandry and Veterinary Sciences, Sindh Agriculture University, Tandojam, Pakistan

**Keywords:** butyrate, rumen epithelium, gene expression, cell cycle, cell apoptosis

## Abstract

In our previous study, we demonstrated that butyrate induced ruminal epithelial growth through cyclin D1 upregulation. Here, we investigated the influence of butyrate on the expression of genes associated with cell cycle and apoptosis in rumen epithelium. Goats (*n* = 24) were given an intra ruminal infusion of sodium butyrate at 0.3 (group B, *n* = 12) or 0 (group A, *n* = 12) g/kg of body weight (BW) per day before morning feeding for 28 days and were slaughtered (4 goat/group) at 5,7 and 9 h after butyrate infusion. Rumen fluid was analyzed for short chain fatty acids (SCFAs) concentration. Ruminal tissues were analyzed for morpho-histrometry and the expressions of genes associated with cell cycle and apoptosis. The results revealed that the ruminal butyrate concentration increased (*P* < 0.05) in B compared to group A. Morphometric analysis showed increased (*P* < 0.05) papillae size associated with higher number of cell layers in epithelial strata in B compared to A. Butyrate-induced papillae enlargement was coupled with enhanced mRNA expression levels (*P* < 0.05) of cyclin D1, CDK2, CDK4, and CDK6 (G_0_/G1 phase regulators) at 5 h, cyclin E1 (G1/S phase regulator) at 7 h and cyclin A and CDK1 (S phase regulators) at 9 h post-infusion compared to A group. In addition, the mRNA expression levels of apoptotic genes, i.e., caspase 3, caspase 9 and Bax at 5 h post-infusion were upregulated (*P* < 0.05) in group B compared to group A. The present study demonstrated that butyrate improved ruminal epithelial growth through concurrent and time-dependent changes in the expressions of genes involved in cell proliferation and apoptosis. It seems that the rate of proliferation was higher than the apoptosis which was reflected in epithelial growth.

## Introduction

Short chain fatty acids (SCFA) are generated in the rumen by microbial fermentation of dietary carbohydrates. Absorption of SCFA across the epithelium contributes 70–80% of the total energy requirement of the ruminants. Acetate, propionate and butyrate are the main SCFA in rumen fluid (Bergman, 1990; Nozière et al., 2000) which induces morpho-functional alteration in ruminal papillae (Gäbel et al., 1991a,b; Krehbiel et al., 1992; Nozière et al., 2000). Conversely, an increase of SCFA absorption has been linked to the surface area enlargement of ruminal papillae in ruminants fed high concentrate diets (Dirksen et al., 1984; Gäbel et al., 1991a,b). The latter could be ascribed to an increased abundance of gene transcripts related to epithelial proliferation (Penner et al., 2011). Butyrate is a four-carbon SCFA normally produced in the smallest ratio of 5–15% in rumen (Bergman, 1990; Nozière et al., 2000). It is preferred metabolic fuels for rumen epithelium and concerned with mucosal health maintenance (Hamer et al., 2008; Leonel and Alvarez-Leite, 2012). The inner wall of the rumen is covered with rumen papillae which increases the surface area for absorption of nutrients (Malhi et al., 2013). The morphological and functional changes in ruminal papillae can be largely ascribed to butyrate (Krehbiel et al., 1992; Shen et al., 2005). Previous studies indicated that sodium butyrate modulates epithelial growth through cell proliferation, differentiation and apoptosis in the small intestine of piglets and stomach of calves and goats (Kotunia et al., 2004; Guilloteau et al., 2009; Malhi et al., 2013). Butyrate improved papillae size and thereby increased surface area in the dorsal sac of the rumen in bulls (Kowalski et al., 2015). Increasing luminal butyrate concentration either by enhancing its endogenous production through dietary manipulation (Guilloteau et al., 2010) or by exogenous administration (Sakata and Tamate, 1978; Shen et al., 2005) has been shown to enhance ruminal epithelial proliferation. The proliferative effects of butyrate differ not only with the age and physiological status of the animals but also with the dose, route and rate of butyrate administration. In young calves and starved adult sheep butyrate induces epithelial proliferation at high doses > 2.5 g/kg of BW/day (Sakata and Tamate, 1978; Mentschel et al., 2001) and in fed cattle at low doses < 0.5 g/kg of BW/day (Shen et al., 2005). Furthermore, the rapid infusion of butyrate induced proliferation whereas the slow infusion method did not (Sakata and Tamate, 1978; Mentschel et al., 2001). In addition to cell proliferation, apoptosis is a vital process involved in the maintenance of the cell homeostasis and modulates the organ growth. Besides rapid proliferation, butyrate also stimulates the apoptosis in order to maintain the normal cell homeostasis (Mentschel et al., 2001). Oral administration of butyrate reduced apoptosis in ruminal epithelium of calves. In colonic epithelial cell culture, the butyrate deprived medium reduced cell number in each phase of cell cycle, with the maximum reduction in G1 phase caused by apoptosis (Luciano et al., 1996). *In vivo* butyrate administration exerts selective effects on cells depending on their phenotypes. Butyrate increased proliferation of normal intestinal cells in piglets (Kien et al., 2008) whereas inhibited proliferation and increased apoptosis in artificially induced-tumor cells (aberrant crypt foci, ACF) in rat intestine (Clarke et al., 2012). It seems that butyrate stimulates apoptosis only in unwanted or defective cells produced during rapid proliferation.

Though the considerable studies have been carried out to evaluate the influence of butyrate on ruminal epithelial growth but the underlined molecular mechanism particularly in reference with cell cycle and apoptosis has not been fully discovered yet. In continuation with our previous study, in which we showed that butyrate induced ruminal epithelial growth is associated with cyclin D1 upregulation, the present study was intended to explore further the influence of butyrate on expression of genes involved in cell cycle and apoptosis.

## Materials and Methods

The experimental plan and procedures were approved by Animal Care and Use Committee for Livestock at Nanjing Agricultural University following the requirements of the regulations for the administration of affairs concerning experimental animals under the “The State Science and Technology Commission of China, 1988” act.

### Experimental Design, Goat Management, and Infusion Method

Twenty four rumen-fistulated goats (Boer × Yangtze River Delta White) of approximately 4 months of age; 18.29 ± 0.64 kg of body weight (BW) at the start of the experiment were used in this study. The goats were housed in individual cemented floor pens (1.2 m × 1.0 m) and were randomly divided into two groups, i.e., A and B (*n* = 12 each). Animals in group B were given intraruminal infusion of sodium butyrate (Merck, Hohenbrunn, Germany) at the dose rate of 0.3 g/kg of BW added in 50 ml of 0.1 mol/l potassium phosphate buffer. Whereas animals in group A received the same amount of buffer without butyrate. The butyrate infusion was performed 1 h before morning feeding within 10–15 s. Animals were fed concentrate (200 g) two times daily in 2 equal meals at 8:00 am and 5:00 pm and provided with free access to hay and water. **Table 1** depicts all of the ingredients in the feed and their chemical compositions. Body weight of goats was recorded weekly. The duration of the experiment was 28 days.

**Table 1:** Chemical composition of diets fed to goats.

Chemical composition	Concentrate^†^	Hay
DM (%)	87.7	89.8
CP (% of DM)	20.8	7.3
Crude fat (% of DM)	3.6	2.0
Crude fiber (% of DM)	6.6	28.2
Crude ash (% of DM)	7.7	6.4
ME (MJ/kg of DM)	10.8	6.9

*^†^The concentrate was composed of ground corn, soybean meal, cottonseed bran, wheat bran, fish meal, calcium phosphate, limestone, trace mineral salt, and vitamin premix (vitamins A, D, and E). DM, dry matter; CP, crude protein; ME, metabolizable energy; MJ, Megajoule.*

Sampling and chemical analysis of the feed was performed on days 7 and 28. On day 14 of infusion trial the ruminal fluid samples were collected in equal portion from atrium ruminis, ventral rumen and caudal dorsal and caudal ventral regions by suction through inserting a pipe and combined to form one sample at 0, 1, 2.5, 3.5, 5, and 7 h post-infusion. The pH was determined by digital pH meter and then the samples were immediately strained through 4 layers of gauze. Afterwards, 1 ml of 5% HgCl_2_ solution was added to 20 ml of rumen fluid and preserved at -20°C for SCFA analysis. Goats were slaughtered on day 28 (4 goats from each group) at three different time points, i.e., 5, 7, and 9 h after last infusion. After slaughter, the abdomens were immediately opened, and the stomachs were exteriorized. After emptying and washing with PBS (pH 7.4), ruminal tissues (approximately 2 cm^2^) from the atrium ruminis, ventral rumen sac and ventral blind sac were taken for morphological analyses of the papillae. These tissues were fixed in 4% paraformaldehyde solution. Epithelium for RNA extraction was taken from ventral blind sac (∼10 g). After cleansing with ice-cold PBS, the epithelium was detached from muscle layer by hand, shifted into liquid nitrogen and then stored at -80°C until analyzed for PCR.

### Determination of Ruminal SCFA Concentration

The SCFA concentrations in the ruminal fluids were detected using a HP6890N chromatograph (Agilent Technologies, Wilmington, DE, United States) according to a standard method (Yang et al., 2012). The carrier gas was nitrogen (99.99% purity) and was applied at a steady flow rate of 2.8 ml/min with a split ratio of 1:30. The capillary column temperature was fixed at 140°C for 4 min and then increased at 25°C/min to 240°C. Regarding the injection port and the flame-ionization detector (FID), the temperatures were set to 180 and 250°C, respectively. Crotonic acid (CH_3_CH) was used as the internal standard.

### Morphometric and Histomorphometric Analyses

One centimeter square piece of paraformaldehyde fixed epithelium, taken from each of the anterior, ventral and ventral blind sac of the rumen was used for morphological analyses of the papillae. Papilla was held gently with forceps and the caliper was slowly opened wide, from base to the tip of papilla to measure the length, and from one side to the other at the mid of papillae to measure the width. The papillae were cut from these epithelial sections and counted (density, number/cm^2^). For every goat in groups B and A, 15 papillae were measured from each of the samples of the atrium ruminis, ventral rumen sac and ventral blind sac. The surface areas of the rumen mucosa (mm^2^/cm^2^) were determined as the length × width × density × 2.

For the histomorphological evaluations, the formalin fixed tissue samples from the atrium ruminis were taken, dehydrated, cleared and embedded in paraffin. Sections of 4-μm thickness were cut and stained by the standard hematoxylin and eosin (H&E) procedure. For each tissue, 25 to 30 papillae were embedded for paraffin-sectioning and microscopic observation. From these, the 4 paraffin sections with the best orientation of papillae in the median sagittal plane were used to evaluate the morphological characteristics of the ruminal papillae by using Image-Pro Plus 6.0 (Media Cybernetics Inc., Bethesda, MD, United States).

For each papillae three visual fields were used for counting cell density from three cell layers of the epithelium, i.e., SS+ SG = the stratum germinativum (SGv) and stratum basale (SB), to reveal the following parameters:

(i) The epithelial cell density of the SGvs in numbers of cells per mm^2^ (n/mm^2^).
(ii) The epithelial cells in SB are in single row and there counting unit is numbers of cells per mm (n/mm).

### Total RNA Extraction, cDNA Synthesis and Quantitative Real-Time PCR

The guanidinium thiocyanate-phenol-chloroform extraction method reported by Chomczynski and Sacchi (2006) was used for the total RNA extraction from the homogenized ruminal tissue. The RNA concentration and integrity were evaluated based on extinction measurements at 260 and 280 nm made with a Biophotometer (Eppendorf, Hamburg, Germany). The absorption ratio (260:280) was between 1.8 and 2.0 for all of the RNA samples, which indicated high RNA purity in every sample. Aliquots of RNA samples were subjected to electrophoresis through a 1.4% agarose-formaldehyde gel to verify integrity. The RNA concentrations in the samples were then adjusted to1 μg/μl. Random hexamer primers (Invitrogen, Shanghai, China) and Moloney murine leukemia virus (M-MLV) reverse transcriptase (Fermentas, Burlington, ON, Canada) were used for the cDNA synthesis. The target genes of interest and their respective sources and primer sequences are listed in **Table 2**.

**Table 2:** Primers used in quantitative real-time PCR analysis.

Gene^†^	Primer sequence 5′ to 3′^‡^	Accession number^§^	Size(bp)^¶^
CCND1	GGTCCTGGTGAACAAACTC	EU525165.1	114
	TTGCGGATGATCTGCTT		
CDK4	TGAGCATCCCAGTGTTGT	NM-001127269.1	122
	CCTTGTCCAGATACTTCCT		
CDK6	AGAGTGATTGCAGCTTTATGTCCA	GAAI01006376.1	158
	TGCCCAGGTTGCTCACTTC		
CCNE1	GGGACAAGCACCTTATGCAAC	NM-001192776.1	153
	GTGTTGCCATATACCGATCAAAGA		
CDK2	CTGCACCGAGACCTTAAACCTCA	BT020790.1	140
	GCTCGGTACCACAGAGTCACCA		
CCNA	TGGACCTTCACCAGACCTACCT	X68321.1	105
	GTGGGTTGAGGAGAGAAACACC		
CCNB1	AGCGGATCCAAACCTTTGTAGTG	NM-001045872.1	137
	CAATGAGGATGGCTCTCATGTTTC		
CDK1	CCAATAATGAAGTGTGGCCAGAAG	NM-174016.2	164
	AGAAATTCGTTTGGCAGGATCATAG		
p21	AGGGCACGTCTCAGGAGGA	NM_001098958. 1	164
	CAGTCTGCGTTTGGAGTGGTAG		
Bax	TCTGACGGCAACTTCAACTG	NM-173894.1	205
	TGGGTGTCCCAAAGTAGGAG		
Caspase 3	AGCCATGGTGAAGAAGGAATCA	NM-001077840.1	156
	ACCACAGTCCAGTTCTGTGCCT		
Caspase 9	TCCTTTGTTCATCTCCTGCTTG	XM-004013798.1	115
	TTTTCCTTGGCTTGGCTTTG		
Bcl-2	GATGACCGAGTATCTGAACCG	NM-001166486.1	120
	GACAGCCAGGAGAAATCAAACA		
GAPDH	TTGTCTCCTGCGACTTCA	HM043737.1	135
	CCACCACCCTGTTACTGTT		

*^†^CCND1, cyclin D1; CDK4, cyclin-dependent kinase 4; CDK6, cyclin-dependent kinase 6; CCNE1, Cyclin E1; CDK2, cyclin-dependent kinase 2; CCNA, cyclin A; CCNB1, cyclin B1; CDK1, cyclin-dependent kinase 1; p21, cyclin-dependent kinase inhibitor; Bax, Bcl-2-associatedX protein; Bcl-2, B-cell lymphoma 2; GAPDH mRNA, Glyceraldehyde 3-phosphate dehydrogenase ribosomal RNA. ^‡^The first primer listed for each gene is the forward primer and the second primer is reverse primer. ^§^Accession number. ^¶^The base pair of primers.*

The relative gene expression was determined via real-time polymerase chain reaction (PCR) by using the MyiQ2 two-color real-time PCR detection system (Bio-Rad Laboratories, Inc., Hercules, CA, United States). Real-time PCR was performed in a total volume of 20 μl containing 1× iQSYBR Green Supermix (Bio-Rad Laboratories Inc., Hercules, CA, United States), a mixture of the forward and reverses primers (500 nM each), cDNA template (1 ng), and a known amount of sterile water for volume adjustment. An initial cycle of 30 s at 95°C was used to denature the cDNA. Forty PCR cycles involving denaturation at 95°C for 10 s and primer annealing and extension at 55°C for 30 s were then performed. A standard dilution series was used to calculate the amplification efficiencies of all of the primers before performing the PCRs for the experimental samples. The efficiencies of all the primers used were between 97 and 101%. Gene expression was normalized to GAPDH. All samples were run in triplicate. These experiments were repeated twice with 80–90% similarity. Melt curve analyses were performed after all of the PCR analyses. The data were analyzed via PCR array data analyses based on the Δ*C*t method with normalization of the raw data to GAPDH; i.e., ΔCt = *C*t_target_ -*C*t_GAPDH_, where *C*t = the cycle threshold. The relative gene expression values were calculated using the formula 2^-ΔΔ^*^C^*^t^, as explained by (Livak and Schmittgen, 2001). All samples were analyzed in triplicate.

### Statistical Analysis

Data are expressed as means ± SE. Differences with a *P*-value of <0.05 were considered significant. An independent sample *t*-test (two-tailed test) was used to compare the data between two groups. All statistical analyses were performed by using SPSS software (version 13.0.1 for Windows; SPSS, Chicago, IL, United States).

## Results

### Ruminal Short Chain Fatty Acid Concentration

**FIGURE 1:**
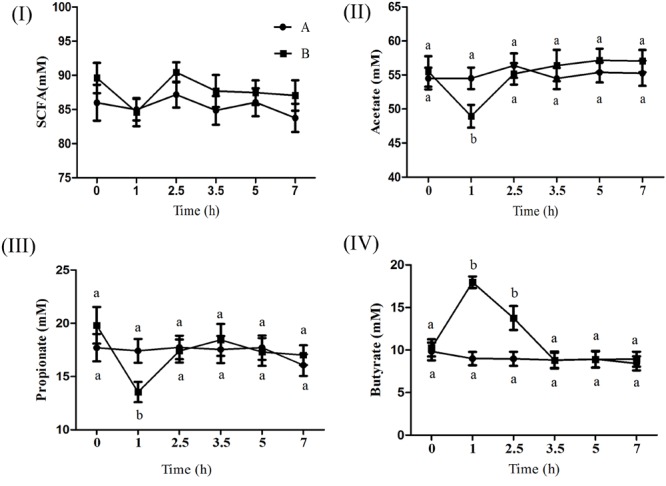
Effect of ruminal butyrate infusion on the molar concentration of total short chain fatty acids **(I)**, acetate **(II)**, propionate **(III)**, and butyrate **(IV)** at 6 time points on day 14th of butyrate infusion. The goats were infused by 0.1 M potassium phosphate buffer (50 ml) with sodium butyrate at 0.3 (B = butyrate group, *n* = 12) or 0 (A = control group, *n* = 12) g/kg of BW per day for 28 days in the experimental duration. Values (means ± SE) differ if they do not share a common letter: ^a,b^*P* < 0.05.

**Figure 1** shows the effect of butyrate infusion on ruminal fermentation pattern of goats. At 0 h the individual and total SCFA concentrations did not differ between the groups, however, at 1 h after butyrate infusion, the molar concentration (mmol/l) of butyrate (17.96) in B increased by about 99.78% (*P* < 0.01) compared to A (8.99). Whereas the molar concentrations (mmol/l) of acetate (48.93 in B vs. 54.50 in A) and propionate (13.54 in B vs. 17.41 in A) were decreased (*P* < 0.05) in B compared to A. The concentrations of both acetate and propionate in group B returned to the base-line levels by 2.5 h whereas the butyrate concentration remained significantly elevated up to 2.5 h and then returned to its base-line value by 3.5 h.

### Morphometric and Histometric Analysis of Rumen Papillae

**FIGURE 2:**
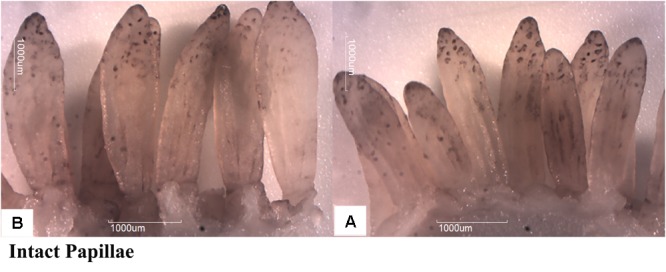
Effect of ruminal butyrate infusion on papillae growth. Goats were infused by 0.1 M potassium phosphate buffer (50 ml) with sodium butyrate at 0.3 (B = butyrate group, *n* = 12) or 0 (A = control group, *n* = 12) g/kg of BW per day for 28 days.

**FIGURE 3:**
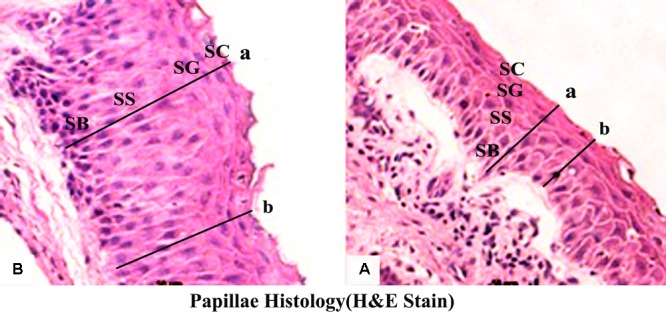
Effect of ruminal butyrate infusion on rumen papillae histology. Goats were infused by 0.1 M potassium phosphate buffer (50 ml) with sodium butyrate at 0.3 (B = butyrate group, *n* = 12) or 0 (A = control group, *n* = 12) g/kg of BW per day for 28 days. Histomicrographs showing epithelial strata at maximum (a) and minimum (b) depths in epithelium of goats. Morphological characteristics of the ruminal papillae observed by using Image-Pro Plus 6.0 (Media Cybernetics Inc., Bethesda, MD, United States).

Butyrate infusion significantly increased (*P* < 0.05) the papillae length, width and density in the atrium ruminis (AR), ventral rumen (VR), and ventral blind sac (VBS) compared to group A (**Table 3** and **Figure 2**). The increases in papillae length, width and density led to an increase (*P* < 0.05) in the surface area of all the tested regions in group B compared to group A. Histometric analysis of rumen papillae revealed that the cell density in the stratum germinativum (SGv) and stratum basale (SB) increased (*P* < 0.05) in group B compared with group A (**Table 4** and **Figure 3**).

**Table 3:** Effect of butyrate on morphometric parameters of rumen papillae of goat^†^.

Parameters	5 h			7 h			9 h		
	Groups		*P*-value	Groups		*P*-value	Groups		*P*-value
	B	A		B	A		B	A	
*Atrium ruminis*									
Length, mm	5.48 ± 0.21	4.50 ± 0.14	0.011	5.21 ± 0.15	4.11 ± 0.11	0.001	5.61 ± 0.13	4.72 ± 0.19	0.011
Width, mm	1.91 ± 0.13	1.41 ± 0.10	0.026	1.94 ± 0.11	1.43 ± 0.10	0.017	2.21 ± 0.14	1.62 ± 0.11	0.016
Density, n/cm^2^	89.25 ± 4.7	71.25 ± 4	0.029	87.50 ± 3.2	75.25 ± 2.8	0.029	88.75 ± 4.3	67.75 ± 3.1	0.010
Surface, mm^2^/cm^2^	1867.33 ± 136	922.24 ± 131	0.002	1785.60 ± 183	889.83 ± 86	0.010	2199.16 ± 129	1028.78 ± 45	0.001
*Ventral rumen sac*									
Length, mm	4.31 ± 0.13	3.62 ± 0.15	0.015	4.31 ± 0.12	3.77 ± 0.14	0.028	4.54 ± 0.18	3.70 ± 0.15	0.013
Width, mm	1.81 ± 0.07	1.40 ± 0.07	0.008	1.81 ± 0.06	1.60 ± 0.04	0.039	1.93 ± 0.13	1.54 ± 0.10	0.057
Density, n/cm^2^	93.00 ± 4.4	78.50 ± 3.7	0.047	91.75 ± 3.7	74.0 ± 2.9	0.010	89.75 ± 3.1	73.0 ± 4.4	0.024
Surface, mm^2^/cm^2^	1449.13 ± 49	803.04 ± 76	0.001	1438.27 ± 110	895.23 ± 57	0.005	1580.03 ± 119	824.34 ± 35	0.001
*Ventral blind sac*									
Length, mm	3.46 ± 0.12	2.70 ± 0.13	0.006	3.22 ± 0.12	2.58 ± 0.15	0.019	3.42 ± 0.14	2.75 ± 0.15	0.020
Width, mm	1.75 ± 0.08	1.30 ± 0.07	0.010	1.70 ± 0.06	1.48 ± 0.04	0.032	1.74 ± 0.15	1.30 ± 0.08	0.054
Density, n/cm^2^	89.75 ± 2.6	77.75 ± 3.8	0.041	90.75 ± 5	74.75 ± 3.90	0.046	85.25 ± 4.2	71.25 ± 4.2	0.059
Surface, mm^2^/cm^2^	1091.93 ± 83	553.21 ± 62	0.002	991.37 ± 52	574.64 ± 41	0.001	1009.02 ± 80	505.92 ± 35	0.001

*^†^Goats were infused by 0.1 M potassium phosphate buffer (50 ml) with sodium butyrate at 0.3 (B = butyrate group) or 0 (A = control group) g/kg of BW per day for 28 days. Values are mean ± SE.*

**Table 4:** Effect of ruminal butyrate infusion on cell density of rumen epithelial strata in goats^†^.

Parameters	5 h			7 h			9 h		
	Groups		*P*-value	Groups		*P*-value	Groups		*P*-value
	B	A		B	A		B	A	
SS+ SG, n/mm^2^	9148.18 ± 291	7936.03 ± 186	0.010	9096.93 ± 264	7797.93 ± 125	0.010	8047.62 ± 258	6883.95 ± 197	0.012
SB, n/mm	292.04 ± 13.2	250.83 ± 4.9	0.027	293.87 ± 15.0	246.58 ± 4.8	0.042	283.75 ± 12.4	242.52 ± 4.1	0.039

*^†^Goats were infused by 0.1 M potassium phosphate buffer (50 ml) with sodium butyrate at 0.3 (B = butyrate group, n = 4) or 0 (A = control group, n = 4) g/kg of BW per day for 28 days. SS, stratum spinosum; SG, stratum granulosum; SB, stratum basale. Values are mean ± SE.*

### Gene Expression Analyses

**FIGURE 4:**
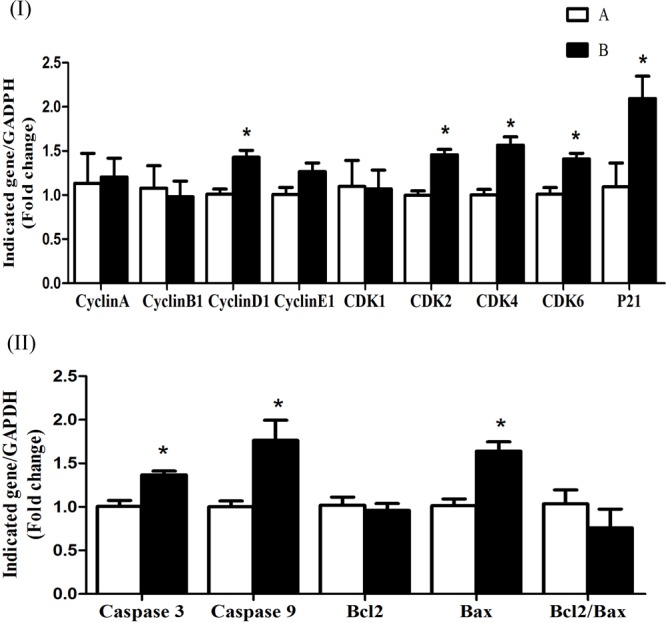
Effect of ruminal butyrate infusion on mRNA expression of **(I)** proliferation and cell cycle inhibition and **(II)** apoptosis related genes in the rumen epithelium of goats. Goats were infused by 0.1 M potassium phosphate buffer (50 ml) with sodium butyrate at 0.3 (B = butyrate group, *n* = 4) or 0 (A = control group, *n* = 4) g/kg of BW per day for 28 days and slaughtered on day 28 at 5 h after infusion. The levels of gene expression were calculated with real-time PCR in comparison with GAPDH mRNA. Values are mean ± SE. Asterisks exhibit the differences between groups with ^∗^*P* < 0.05.

**FIGURE 5:**
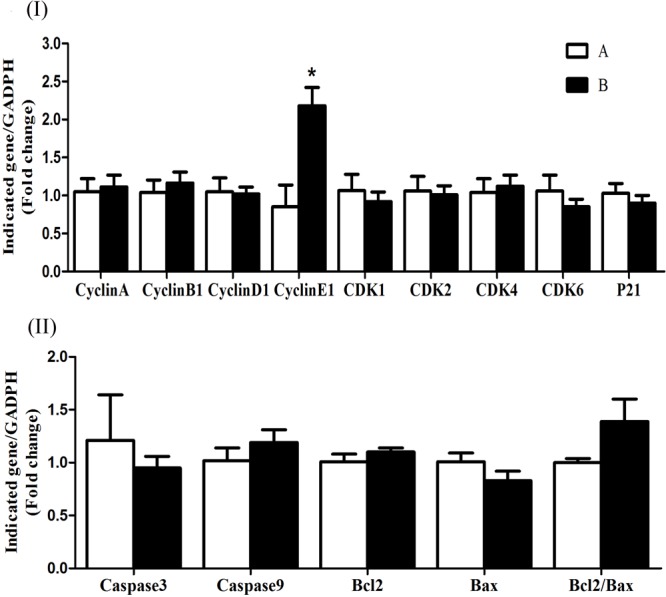
Effect of ruminal butyrate infusion on mRNA expression of **(I)** proliferation and cell cycle inhibition and **(II)** apoptosis related genes in the rumen epithelium of goats. Goats were infused by 0.1 M potassium phosphate buffer (50 ml) with sodium butyrate at 0.3 (B = butyrate group, *n* = 4) or 0 (A = control group, *n* = 4) g/kg of BW per day for 28 days and slaughtered on day 28 at 7 h after infusion. The levels of gene expression were calculated with real-time PCR in comparison with GAPDH mRNA. Values are mean ± SE. Asterisks exhibit the differences between groups with ^∗^*P* < 0.05.

**FIGURE 6:**
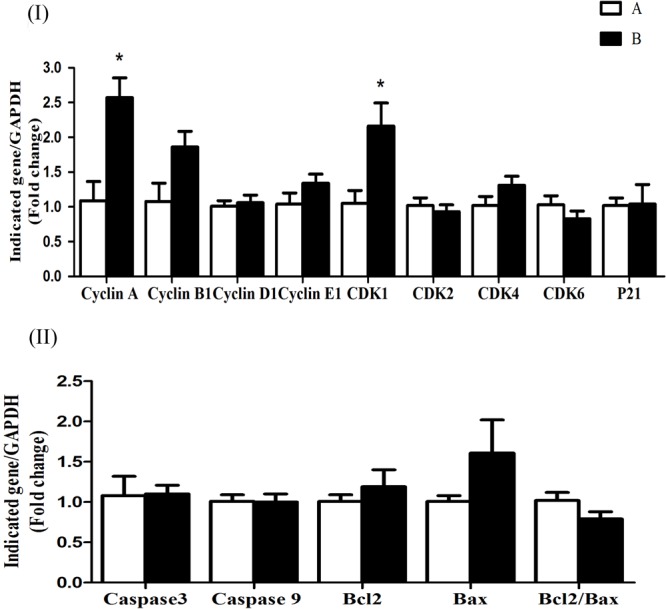
Effect of ruminal butyrate infusion on mRNA expression of **(I)** proliferation and cell cycle inhibition and **(II)** apoptosis related genes in the rumen epithelium of goats. Goats were infused by 0.1 M potassium phosphate buffer (50 ml) with sodium butyrate at 0.3 (B = butyrate group, *n* = 4) or 0 (A = control group, *n* = 4) g/kg of BW per day for 28 days and slaughtered on day 28 at 9 h after infusion. The levels of gene expression were calculated with real-time PCR in comparison with GAPDH mRNA. Values are mean ± SE. Asterisks exhibit the differences between groups with ^∗^*P* < 0.05.

The mRNA expression levels of cyclin D1, CDK4, CDK6, CDK2, and p21 were significantly increased (*P* < 0.05) in group B compared to A at 5 h after butyrate infusion (**Figure 4I**). Whereas the butyrate infusion increased (*P* < 0.05) the mRNA expression levels of cyclin E1 at 7 h and that of cyclin A and CDK1at 9 h post-infusion compared to A (**Figures 5I, 6I**). The assessment of genes showed that the butyrate infusion caused significant increase (*P* < 0.05) in mRNA expression levels of caspase 3, caspase 9 and Bax at 5 h post-infusion compared to control (**Figure 4II**). However, the expression levels of apoptotic genes at 7 and 9 h post-infusion were remained unchanged (*P* > 0.05) between the groups (**Figures 5II, 6II**). Furthermore, the increased mRNA expression levels of cyclin D1, cyclin E1, CDK2, 4, and 6, and P21, caspase 3, caspase 9 and Bax were positively correlated with the ruminal butyrate concentration in B and the differences were significant (*P* ≤ 0.05, **Table 5**).

**Table 5:** Correlation between ruminal butyrate and mRNA concentration level of genes.

Item	*r*	*P*-value
Butyrate × Cyclin A	0.146	0.730
Butyrate × Cyclin B1	–0.048	0.910
Butyrate × Cyclin D1	0.773	0.025
Butyrate × Cyclin E1	0.713	0.047
Butyrate × CDK1	0.075	0.859
Butyrate × CDK2	0.864	0.006
Butyrate × CDK4	0.954	0.000
Butyrate × CDK6	0.773	0.024
Butyrate × P21	0.892	0.003
Butyrate × Caspase 3	0.789	0.020
Butyrate × Caspase 9	0.927	0.001
Butyrate × Bax	0.863	0.006
Butyrate × Bcl-2	–0.028	0.947

*Goats were infused by 0.1 M potassium phosphate buffer (50 ml) with sodium butyrate at 0.3 (B = butyrate group) or 0 (A = control group) g/kg of BW per day for 28 days. mRNA concentration was analyzed for correlation with ruminal butyrate concentration. (r) = measure of the strength of the association between butyrate concentration and mRNA of genes.*

## Discussion

### Fermentation Pattern and Morphology of Rumen

Butyrate concentration in rumen fluid of B group increased from 1 to 2.5 h post-infusion compared to A group and declined subsequently. Conversely the molar concentration of acetate and propionate significantly decreased at 1 h post-infusion. The changes in the concentration of three major ruminal SCFAs in response to intraruminal infusion of butyrate are consistent with previous study (Malhi et al., 2013). The reduction of acetate and propionate might be due to inhibitory impact of butyrate on their production by ruminal microbes (Li et al., 2012) or butyrate could promote the absorption of acetate and propionate by the ruminal epithelium (Malhi et al., 2013). In the present study the ruminal infusion of butyrate increased dimensions and density of papillae in atrium ruminis, ventral sac and ventral blind sac of rumen. Furthermore, the histometric analysis showed that the number of cell layers in SGv and density of cells in SB increased in butyrate infused goats compared to control. This could be largely attributed to increased intraruminal butyrate concentration as previously reported by (Mentschel et al., 2001; Shen et al., 2005; Malhi et al., 2013).

### Genes Expression Related to Proliferation and Apoptosis

In the present study, the butyrate infusion showed time-dependent effects on genes related to cell cycle and apoptosis in ruminal epithelium. The mRNA expressions level of cell cycle genes involved in early to mid G1 phase, i.e., cyclin D1, CDK2, CDK4, and CDK6 increased at 5 h and those involved in late G1 phase, i.e., cyclin E1 at 7 h after butyrate infusion. Based on histo-morphometric studies, many researchers have demonstrated that butyrate improves rumen epithelial growth through increased cell proliferation (Sakata and Tamate, 1978; Mentschel et al., 2001; Shen et al., 2004). The cell cycle of a mammalian cell including ruminal epithelium is a complex process consisting of four phases including G1, G2, S, and M (Goodlad, 1981; Mathew et al., 2010). In general, the rumen epithelial cells complete the cell cycle in a period of 24 h, however, the duration may increase or decrease depending upon shortening or elongation of various cell cycle phases under the influence of diet or dietary-produced SCFA (Goodlad, 1981). The cell cycle phases are strictly regulated by cyclins and cyclin-dependent kinases (CDK) proteins (Matsushime et al., 1992; Jacks and Weinberg, 1996). Cyclin D1 forms a complex with either CDK4/6 that promotes the cells from early to mid G1 phase (Mathew et al., 2010). The upregulation of cyclin D1 and CDK 4/6 at 5 h after butyrate infusion suggest that butyrate enhanced the transition of cells through G_0_/mid-G1 phase. The cyclin E1 mRNA synthesis is initiated during the G1 phase and its expression reaches at its peak in late G1 (Petersen et al., 1999; Coverley et al., 2000). In the present study, the increased cyclin E1 mRNA expression level in ruminal epithelial tissue collected at 7 h post-infusion suggest that the cell population was in G1/S transition phase. If we presume that at the time of butyrate infusion (0 h) the ruminal epithelial cells were in G_0_-phase, the cells took 4–5 h to enter mid-G1 phase and another 2–3 h to enter the mid/late G1 phase and the total duration of G1-phase would be 6–7 h. In sheep fed roughage-based diet, the maximum duration of G1 phase in rumen epithelial cells was 8.2 h which reduced to 6.5 h when the sheep were transitioned from roughage to concentrate based-diet (Goodlad, 1981). Previous studies have shown that butyrate induces ruminal epithelial growth in goats by shortening G1 phase through cyclin D1 upregulation (Malhi et al., 2013). Moreover, the cyclin D1 expression was influenced by sampling time. According to our previous report, ruminal tissue samples collected at 3 h post-infusion of butyrate already showed significant increase in cyclin D1 mRNA expression level compared to control whereas at 7 h post-infusion did not (Malhi et al., 2013). In the present study, we also observed that cyclin D1 expression was remained elevated at the ruminal tissue samples collected at 5 h post-infusion but not at 7 h post-infusion (**Figure 4**). On the other hand, we found significantly increased CDK4 expression (**Figure 4**) but they not observe any change in CDK4 (Malhi et al., 2013). The reason of this contradiction is not clear. To our best knowledge, the influence of *in vivo* butyrate on cyclins A, B and E1, and CDK 1 and 2 and their possible role in ruminal epithelial growth has not been previously reported. However, high concentrate diet-induced ruminal epithelial proliferation was associated with modulation of cell cycle genes including cyclins D1, E1, A and B, and CDK 2/4 and 6 (Gui and Shen, 2016). The activity of cyclin E requires CDK2 with which it forms cyclin E1/CDK2 complex that derives the cell cycle through late G1/S phase (Sherr and Roberts, 1999, 2004). However, in the present study, we did not observe any change in CDK2 mRNA expression. This suggests that there could be an alternate mechanism for binding with cyclin E1 to drive the late G1 events of cell cycle. The loss of CDK2 in mice did not affect G1/S transition and cell cycle progression and it was suggested that either cyclin E1 performed independent of CDK2 (Berthet et al., 2003; Ortega et al., 2003) or possibly formed cyclin E/CDK1 complex (Aleem et al., 2005). Recently, it has been shown that cyclin E1 activates CDK3 and forms cyclin E/CDK3 complex to promote G1/S transition (Satyanarayana and Kaldis, 2009).

Once the cells enter the S-phase the cyclin E1 expression is subsided and the synthesis of cyclin A mRNA is initiated and its expression becomes maximal with the progression of S-phase (Petersen et al., 1999; Coverley et al., 2000). In the present study, the cyclin A mRNA expression increased in sample collected at 9 h post-infusion which suggests that the cell population entered the S-phase. The activity of cyclin A requires either CDK1 or 2 to form the complex cyclin A/CDK1/2 which regulates the transition of cells through S-phase. In the present study, the CDK1 mRNA expression concurrently increased with cyclin A mRNA expression. Finally cyclin B is required prior to mitosis (G2/M-phase) (Petersen et al., 1999; Coverley et al., 2000) which binds with CDK1 to accomplish cell division (Riabowol et al., 1989). In the present study, no significant changes in cyclin B1 mRNA expression were observed in ruminal epithelial tissue. This can be explained by the fact that cell populations in the tissues collected at specific time points in the present study did not arrive at G2/M-phase of cell cycle. However, the tissue samples collected at 9 h showed that butyrate infusion caused slight increase in cyclin B1 expression which indicates the transition of cells through late-S/G2-M phase since the genes required in next phase of cycle initiate to express in late stage of previous phase (Petersen et al., 1999; Coverley et al., 2000). The rise in cyclin B1 expression could be expected if the fourth ruminal tissue sample were collected at certain time-point after 9 h post-infusion. The present data demonstrate that the proliferative effects of butyrate depend upon the specific stage of dividing cells. Thus, while performing *in vivo* experiments the location and tissue sampling time is very important because the tissue sample collected from a particular site at one specific time would represent the cell population in a particular phase of cell cycle with expression of associated genes (Li et al., 2009; Yang et al., 2012).

**FIGURE 7:**
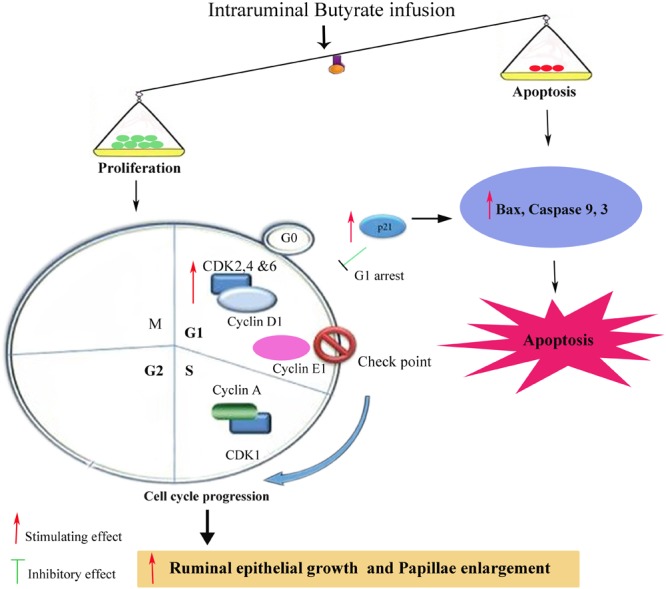
Schematic diagram showing influence of ruminal Sodium Butyrate (SB) infusion on expression of genes related to proliferation and apoptosis in rumen epithelium of goat. SB increased proliferation through upregulation of cell cycle related genes involved in early G1 (cyclin D1, CDK2, CDK4, and CDK6), mid G1 (cyclin E1) and S phase (cyclin A and CDK1). Simultaneously, SB also increased the mRNA expression level of p21, Bax, Caspases 3 and 9, leading to cell cycle arrest and apoptosis. Since, the rate of proliferation was higher than apoptosis which was reflected in ruminal epithelial growth and papillae enlargement.

In the present study, the butyrate infusion increased p21 mRNA expression level in ruminal tissue collected at 5 h post-infusion. The p21 has anti-proliferative effects which binds with CDKs and prevent cell cycle progression and arrest cell cycle at any phase through G0/G2 phases (Ogryzko et al., 1997; Niculescu et al., 1998). It has been shown that the increased proliferation rate in rumen epithelium leads to rise in apoptosis in order to maintain the normal cell homeostasis (Mentschel et al., 2001). Consistent with our findings, (Crim et al., 2008) have shown that *in vivo* butyrate administration upregulated p21 expression in colonocytes of rats. In addition, butyrate-induced p21 upregulation increased apoptosis in artificially induced-tumor cells (aberrant crypt foci, ACF) in rats fed fish oil but no apoptotic effects were observed in ACF in rats fed corn oil. This suggests that apoptotic effects of butyrate depend upon the associated factors and physiological status of the cell even after p21 upregulation. Many studies have shown such selective effects of *in vivo* butyrate which depend upon physiological status and cellular phenotypes. Cecal infusion of butyrate increased proliferation of normal intestinal cells in piglets (Kien et al., 2008) whereas butyrate inhibited proliferation and increased apoptosis in artificially induced-ACF in rat intestine (Clarke et al., 2012). The results of present study demonstrate that butyrate induced apoptosis in ruminal epithelial cells during rapid proliferation, since the overall ratio of proliferation was higher than the apoptosis which was reflected in epithelial growth (**Figure 7**).

For normal growth and tissue homeostasis a balance between cell proliferation and apoptosis is important. Depending on the cellular context these two antagonistic processes are linked via shared molecular machinery (Evan et al., 1995). In the present study, the butyrate infusion caused upregulation of proapoptotic genes, i.e., Bax, caspase 3 and caspase 9 in ruminal epithelial tissues at 5 h post-infusion, however, the expressions of apoptotic genes at 7 and 9 h were not influenced by the butyrate treatment. This suggests that butyrate induced apoptosis in ruminal epithelial cells by arresting cell cycle at G1. The inhibitory effects of butyrate on G1 phase of cell cycle and the induction of apoptosis have been well established (Li and Elsasser, 2005; Li and Li, 2006; Mathew et al., 2010). As mentioned earlier that p21 is also upregulated at the same time point which suggests the involvement of p21 pathway in butyrate-induced apoptosis. Consistent with our findings, previous studies have shown that *in vivo* butyrate administration increased apoptosis through p21-dependent pathway in prostate cancer cell lines implanted in mice (Kuefer et al., 2004). Over expression of caspase 3, caspase 9, and Bax in the present study indicate that butyrate involved p21-dependent intrinsic pathway mediated by Bax and caspase 3. In prostate cancer cells butyrate treatment up-regulated Bax (Mu et al., 2013).

## Conclusion

The present study demonstrates that butyrate stimulates epithelial growth by modulating both proliferative and apoptotic genes. Butyrate enhanced ruminal epithelial proliferation by shortening various cell cycle phases through associated cyclins/CDKs upregulation. Besides proliferation, butyrate induced apoptosis in rumen epithelium through activation of Bax, caspase 3, and caspase 9. Since the ratio of proliferation was higher than the apoptosis which was reflected in epithelial growth. To our knowledge, we report for the first time the effects of *in vivo* butyrate infusion on cell cycle and apoptotic genes in rumen epithelium of goats. Moreover, the environment of large intestine in mammals generally resembles with the environment of rumen so the results of present study can be extrapolated on mammals including human beings. However, the results of present study may be carefully interpreted as rumen epithelium is a complex tissue consisting of four different types of cell layers and the effects of butyrate vary with cell phenotypes. Nevertheless, this study provides a basis for understanding the molecular mechanism underlying the butyrate action on ruminal epithelial growth.

## Author Contributions

JS wrote the paper. ZS conceived and designed the study. HG and BZ acquired the data. JS, HG, ZL, and BZ analyzed and/or interpreted the data. ZS, HG, and ZL drafted and revised the manuscript critically for important intellectual content. All authors read and approved the final manuscript.

## Conflict of Interest Statement

The authors declare that the research was conducted in the absence of any commercial or financial relationships that could be construed as a potential conflict of interest.
